# Practices improvement of building information modeling in the Egyptian construction projects

**DOI:** 10.1038/s41598-024-63357-5

**Published:** 2024-06-12

**Authors:** Yasmin Nabil, Ahmed H. Ibrahim, Suad Hosny

**Affiliations:** Department of Construction Engineering and Utilities, Faculty of Engineering, Zagazig University, Zagazig, Egypt

**Keywords:** Reinforced concrete (RC), Building information modeling (BIM), Construction projects (CP), Construction industry (CI), Reducing reinforced concrete material waste (RRCMW), Engineering, Materials science, Mathematics and computing

## Abstract

Reduced Reinforced Concrete Material Waste (RRCMW) in building projects is regarded as a critical issue that must be managed. The main purpose of the research is to illustrate the importance of BIM in construction. Also, it is found that the main objectives of this paper are to study the improvement of practicing BIM in Egypt and, practicing of BIM in construction industry in Egypt is also measured. Two questionnaires survey are conducted. The first questionnaire is to measure the improvement of using BIM during the last 7 years and it is discovered that there is a massive improvement in using BIM in this period. The second questionnaire is to determine the adopting value of BIM in Egyptian projects in order to meet the study objective. So, based on the questionnaire analysis, it is discovered that about 94% of consultants actually practicing BIM in 3D while about 72% of contractors agree with practicing BIM in 3D. Also it is found that about 86% and 78% of consultants actually practicing BIM in 4D and 5D while only about 43% and 40% of contractors agree with practicing BIM 4D and 5D model respectively. Only about 61% and 58% considered that BIM is important in 6D and 7D respectively because it isn’t widely used in Egypt and engineers use BIM up to 5D. As a result, the findings reveal that the number of consultant’s site engineer’s respondents are more than contractors because the usage of BIM is effective in the field of design and consultancy more than using in site and while BIM isn't extensively utilized in Egypt, engineers should be familiar with it because it will be a useful tool in the future. So, the main purpose of this study is to illustrate practicing of BIM in the Egyptian construction projects and study the improvement of using BIM during the last 7 years in Egypt because BIM is considered as an important technology used to reduce waste in construction projects from design stage to construction and operation stage but still not used in Egypt in a wide range till now, so it is very crucial to study this issue. Also, another main objective of this study is to compare the development done in using BIM during the last 8 years to make sure that using BIM in Egypt is going on and developed.

## Introduction

Waste in construction sites is because of a lack of the attention being paid to the size of product used, lack of the interest of contractors and lack of knowledge about construction during activities^1^. To diagnose the nature of the Egyptian Construction Industry, a previous study performed by Garas et al.^2^ is considered. This study aims to identify the main causes affecting materials waste in Construction Egyptian Industry. It is found that main causes of material waste are late information, uncompleted design, inadequate information, poor control, unnecessary people move, untrained labor, work not done, poor technology of equipment, changes in design and damage during transportation. So, it is very critical to study this issue in Egypt. According to Shaqour and Almashhour^3^ the most common causes of waste in Egypt are selecting contractors with low experience, material damage in construction sites, poor planning and scheduling of the work by contractors, selection of low quality material, poor storages, and poor control on work contractors. Also, construction and demolition waste is a critical issue in Egypt. The problem is serious in Egypt, in which CDW represent up to 40% of total materials cost in construction projects and the main factors behind CDWG in Egypt can be summarized as follows: (1) deficiencies in waste-efficient practices; (2) lack of awareness; (3) absence of appropriate culture and behaviour; (4) lack of strict legislation; (5) lack of coordination among project parties; (6) scar city of legal dumpsites; and (7) lack of adoption to CDW recycle and reuse^4^. Also, a study conducted by Reda et al.^5^ to investigate the applicability and effectiveness of Safe Disposal of Construction Demolition Waste (SDCDW) factors in the Egyptian construction industry and examining the relationship between these factors and SDCDW found that (1) among the various factors, ‘‘selection of the shortest path transport route” has the highest applicability, while ‘‘detection of CDW illegal disposal sites” is the most effective factor; and (2) there exist positive statistical relationships between SDCDW and all of the various factors.

**Figure 1:**
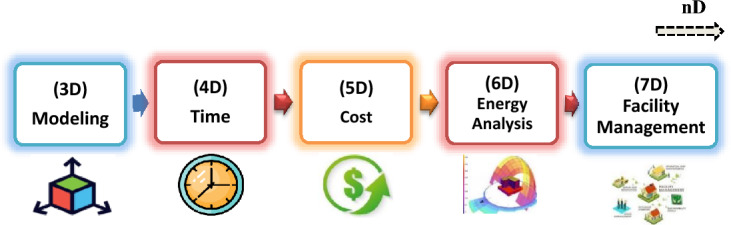
BIM dimensions (evolutions).

Building Information Modeling (BIM) is a new revolution in our daily construction industry which represents the most important method to calculate all the components of any construction project accurately. The large amount of waste and in efficiency is because on-site rework as every change order that cost the owner or the builder money and don’t add value to the building is considered as waste. To avoid or reduce the construction waste, procedures that is based on careful planning, monitoring and controlling system should be applied^6^. So using building information modeling (BIM) is the most appropriate technique to use. BIM plays an important role in reducing waste during the design and pre-construction phase and involves representing a design as objects that carry their geometry, relations, and attributes^7^. Although not called BIM, the original concepts of BIM have been developed since 1970^8^. It was also found that about 70% to 80% of drawing are currently produced digitally in Scandinavian construction sector^9^. At the past the traditional methods were used for drawing sections, elevations, and plans by using ruler and papers then using the 2D program as AutoCAD (2D) appeared. The main difference between 2D CAD and BIM is that 2D CAD describes building as plans, sections, and elevations and editing one of these views requires checking and update all the other views. Another main difference is that using the traditional method 2D CAD represents the building as lines, arcs, and circles while objects are defined in BIM as spaces, walls, beams, and columns. BIM includes all information, quantities, and properties of building elements, cost estimates, materials inventories and project schedule and demonstrates the entire building life cycle, so results can be extracted from BIM model easily and accurately^10^. Figure 1 demonstrates BIM dimensions.

To protect existing natural resources, building waste must be managed properly, which necessitates comprehensive training on the identification and separation of recyclable materials at construction sites^11^. One of the key reasons of waste creation has been identified as a lack of on-site waste management strategy^12^. Without a forecast of construction waste quantities related to the project execution plan, proper execution and planning are impossible^13^. Building Information Modeling (BIM) is a new construction industry revolution that represents the most significant way for correctly estimating all the components of any building project. On-site rework is responsible for the enormous amount of waste and inefficiency. Every change order that costs the owner or builder money but adds no value to the project is considered waste. Procedures based on monitoring, controlling systems, and careful planning should be used to minimize or decrease construction waste^6^. As a result, the most appropriate strategy is to apply building information modelling (BIM). BIM is a technique for reducing waste in building projects throughout the design and pre-construction phases. It includes representing a design as objects with relationships, geometry, and properties^7^. BIM contain all information, quantities, and properties of building elements, as well as material inventories, cost estimates, and project schedules, and represents the whole building life cycle, allowing for easy and accurate extraction of results from the BIM model^10^. Building Information Modeling (BIM) consider as the most important new technique in the construction industry. A study illustrates by Autodesk^14^ demonstrates that 42% of non-users of BIM believe that BIM will be highly or very highly important in 5 years, and also found that the growth of BIM in use raised from 28% in 2007 to 48% in 2009. Another study is conducted by McGraw Hill Construction^15^ find that the adoption of BIM in US expands from 49% in 2009 to over 71% in 2012 and about 15,000 h across the whole design has been saved according to WRAP UK Construction^16^. BIM also provides accurate visualization for all project's members to have a complete understanding of materials and machinery layout, activity procedure and conflicts between building elements^6^. BIM can be classified into Design BIM and Construction BIM. Design BIM demonstrates the coordination within the design team (the architect, structural, and mechanical engineer) and Construction BIM related to the actual fabrication and construction of materials to 100% accurate dimensioning. Design and construction BIM have valuable uses and are improving the overall quality and economy of construction, so integrated BIM can be defined as the process that removes waste and created additional value by reducing construction schedule, costs and risks^17^. So, the main purpose of this study is to demonstrate practicing of BIM in the Egyptian construction projects and study the improvement of using BIM during the last 7 years in Egypt.

## Practicing of BIM in construction projects

There are several benefits of BIM, the main benefit is how to create the model virtually and check its constructability in real world before it is done actually. This allows for more efficiency and better designed structures that limit waste of resources. It also allows for improved planning and scheduling, helps to reduce cost and allows for a better collaboration on the job site^6^. One of the most important benefits of BIM are: making early analysis of design at the design phase and allowing to quickly transport very accurate information^14^. BIM aims to develop deep learning about fundamental of structural analysis and design, improve digital modeling, and achieve quality to meet today's demands^18^. BIM also could enhance communication, increase efficiency and reduce errors which helps to reduce resources, energy, materials and waste and provides the opportunity of testing, revising, rejecting and accepting design ideas in real-time^19^. The following Table 1 illustrates practicing BIM in construction projects.

**Table 1:** Practicing BIM in construction projects.

Author name	Year	Practicing BIM in construction projects
Azhar et al.^20^	2011	Discusses the benefits and possible risks of BIM and future challenges for the construction industry. First presented is the main concept of BIM with its advantages and possible applications in construction. Then the role of BIM in the construction industry and academia is discussed based on the results of three questionnaire surveys
Ahankoob et al.^6^	2014	Discusses current waste reduction practices at construction sites with regard to material waste and introduces some measures that are performed to reduce the impact of material waste, as well as the potential of building information modelling as a coordination tool to support individuals in achieving their goals through a more efficient and sustainable vision
Li et al.^21^	2014	Focuses on the quantitative quantification (using a case study methodology) of BIM’s advantages over conventional non-BIM technologies in terms of building construction resource management and real-time cost control through the dynamic querying and statistical analysis of construction schedules, engineering, resources, and costs using BIM technology
Cheng and Kumar^22^	2014	It is recommended to use BIM technology to create an automated framework to generate dynamic site layout models. A dynamic layout model for facility layout management may be constructed automatically using information from BIM models and construction schedules
Smith^23^	2015	Examines the practical obstacles and limitations that project cost management experts meet when implementing and effectively using the many tools, technologies, and software options that are now available in the rapidly developing Building Information Modelling (BIM) industry
Sheng et al.^24^	2016	Discusses how 5D BIM offers a high degree of practicability, which distinguishes BIM from Computer Aided Design (CAD by allowing decision makers to have an advanced understanding of information, which is nearly impossible with the traditional 2D CAD process
El-Yamany^32^	2016	Explains how building information modelling (BIM) is now being used and utilized in the Egyptian construction industry. The research’s findings demonstrated that BIM has been expanding throughout the construction industry and that practitioners believe it to be the future as we transition to sustainable buildings
Vitasek and Zak^25^	2018	Describes how to leverage data from information models to estimate costs using the suggested process scheme today. It also specifies what has to be done to fully utilize the BIM approach in terms of cost estimation
Vitasek and Zak^26^	2018	Deals with the production of quantity takeoffs and budgeting for building projects based on an information model. Communication with two global construction businesses operating in the Czech market has enabled the integration of academic knowledge with building practice
Shaqour and Almashhour^3^	2020	Identifying causes of waste and their levels in different building materials in the construction industry in Egypt to select suitable strategies for managing and improving construction processes
Hosny et al.^27^	2022	Investigates the role of BIM in minimizing reinforced concrete material waste in Egypt. According to case studies, adopting BIM is significantly more successful and economical than using the traditional method, because it assists in the resolution of several issues before and during construction, such as clash detection, material storage, material ordering, and so on
Moradi and Sormunen^28^	2023	Fill the mentioned knowledge gap through exploring and comparing the challenges, enablers, techniques as well as benefits of integrating LC with BIM and sustainability in building construction projects
Almujibah^29^	2023	A quantitative case study examines the factors that led to the success of employing the BIM tool in managing a recent home development project in Jeddah, Saudi Arabia

## Research methodology

**Figure 2:**
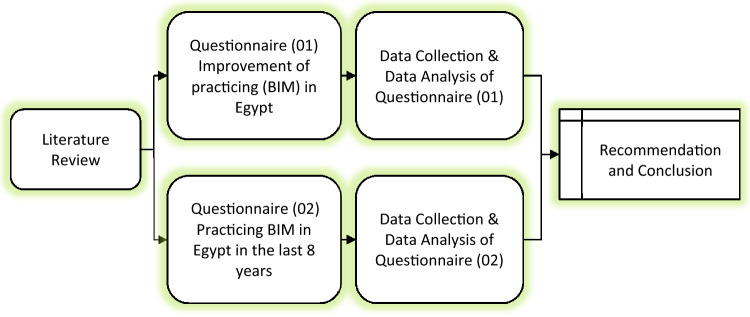
Methodology chart.

Applying social research interviews and questionnaire survey is the most used technique of data collection. The research methodology consists of collected data using a structured questionnaires. The first questionnaire purpose is to study the improvement of BIM in Egypt and, the second questionnaire main objective is to study practicing BIM in Egypt in the last 8 years compared with another Egyptian study. Figure 2 demonstrates the research methodology in detail.

## Questionnaire design

Questionnaires consists of the participant’s personal information as position and experience. For the first questionnaire, improvement of practicing Building Information Modeling in Egypt during the last 7 years is measured, using option out of the 1–5 scale, 1 = “Strongly Agree”; 2 = “Agree”; 3 = “Neutral”; 4 = “Disagree” and 5 = “Strongly Disagree”. For the second questionnaire, some general questions about the importance of BIM are distributed to respondents to analyze the practicing of BIM in construction industry, using option out of the 1–5 scale, 1 = “Highly unimportant”; 2 = “Unimportant”; 3 = “Neutral”; 4 = “Important” and 5 = “Highly important”. Data was put in excel sheets after filling the questionnaire and applied Statistical Package for Social Science (SPSS) for further analysis. The main usage of SPSS is checking the consistency of data. Mean score and SD are measured. To provide a degree of importance, an importance index is calculated as shown in Eq. (1). Where Pi = Practicing index; a_i_ = weight of the ith response; xi = frequency of the ith response; and i = response category index.

In this study, reliability test is conducted to check the data uniformity through (Cronbach Alpha) to check the consistency of data. Generally, the reliability coefficient of 0.7 and above is good and acceptable^30^. So, data for this study is acceptable and reliable. The Cronbach’s Alpha for this study based on SPSS analysis is 81% which considered suitable and acceptable.

1 $$Pi = \sum\limits_{i = 1}^{n} {\frac{{{\text{ai}}*{\text{xi}}}}{5}}$$

According to a study conducted by Baxter and Bartlett^31^, the following formula is used to compute the required sample size for this study.

2 $$P = \frac{{K^{2} *P(1 - P)}}{{E^{2} }}$$

where N is the sample size needed, K value equals 1.645 with confidence level of 90%, P degree of variance is 0.5 with E the acceptable margin of error = 10%. By substituting all of these parameters in previous equation, the required sample size of this study is 68 as a minimum value.

## Analysis and discussion

### Questionnaire respondents

Two questionnaires survey are conducted and distributed to contractor and consultant site engineers from different types of construction projects such as residential and bridges projects. The questionnaires are distributed to 200 respondents in the top, medium and lower-level management working in construction site who deals regularly with reinforced concrete works and who applies BIM practically. Only 180 responds are participating and answering the two questionnaires. So, the total number of participants taken into consideration in this study are 180 and they are distributed as 81 contractor engineers and 99 consultants’ engineers.

**Figure 3:**
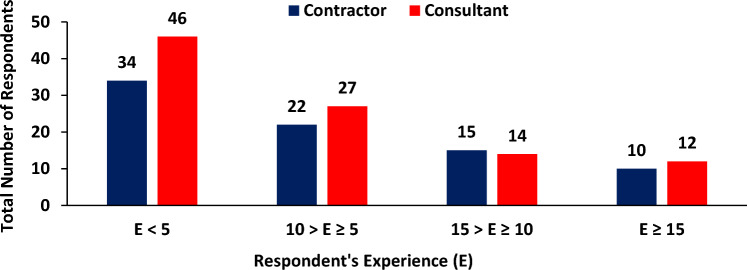
Participants respondents according to experience.

BIM is a very effective tool in minimizing project cost, eliminating design errors and reducing rework. Based on the questionnaire analysis, the respondents are asked about their perspective of applying BIM compared with traditional method. The respondents are categorized into contractor site engineers and consultant site engineers. The number of consultant’s engineer’s respondents are more than contractors because the usage of BIM is effective in the field of design and consultancy more than using in site. Figure 3 illustrates the number of respondents based on their experience (E).

### Analysis and discussion of improvement of practicing building information modeling in Egypt

**Figure 4:**
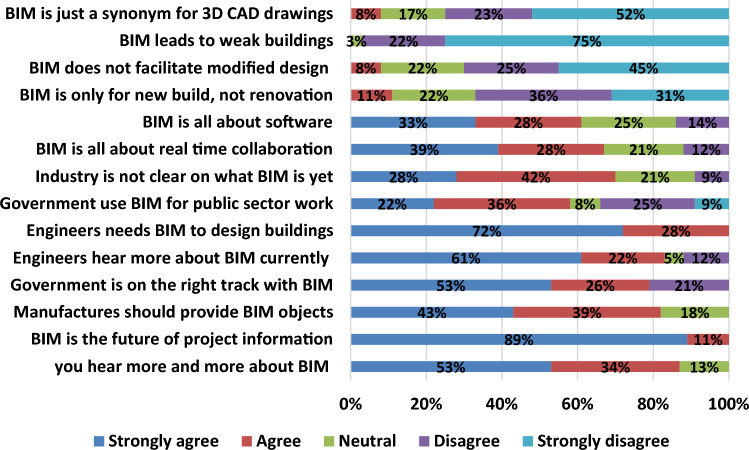
Study the improvement of practicing BIM.

El-Yamany^32^ find that using BIM is limited and not effective in Egypt till now. The research concluded that BIM is expanding in the Egyptian construction industry in the near future and practitioners think it is the future of construction industry while moving to sustainable buildings. The research also concluded that the construction industry don’t have enough knowledge to apply BIM, which enhance the need to provide more knowledge and information to all project participants regarding the benefits of BIM technology. So it is too important to make a questionnaire to measure the improvement of practicing BIM in Egypt. This questionnaire is a new copy from El-Yamany^32^ questionnaire. The purpose of this questionnaire is to compare between the results of respondents about the importance of using BIM in Egypt during the last 7 years. The results of the questionnaire are as shown in Fig. 4 and Table 2.

**Table 2:** Study the improvement of practicing BIM.

Improvement of practicing BIM	Strongly agree (%)		Agree (%)		Neutral (%)		Disagree (%)		Strongly disagree (%)	
	Elyamany, 2016	Research results	Elyamany, 2016	Research results	Elyamany, 2016	Research results	Elyamany, 2016	Research results	Elyamany, 2016	Research results
You hear more and more about BIM	51	53	40	34		13				
BIM is the future of project information	86	89	9	11			5			
Manufactures should provide BIM objects	51	43	33	39		18	16			
Government is on the right track with BIM	14	53	7	26			30	21	49	
Engineers hear more about BIM currently	51	61	40	22		5	9	12		
Engineers needs BIM to design buildings	46	72	42	28			12			
Government use BIM for public sector work	7	22	14	36		8	63	25	16	9
Industry is not clear on what BIM is yet	19	28	51	42	23	21	7	9		
BIM is all about real time collaboration	5	39	51	28	37	21	7	12		
BIM is all about software	5	33	30	28	35	25	30	14		
BIM is only for new build, not renovation	2		12	11	41	22	49	36	16	31
BIM does not facilitate modified design			12	8	26	22	39	25	23	45
BIM leads to weak buildings	2		7		21	3	19	22	51	75
BIM is just a synonym for 3D CAD drawings			7	8	20	17	33	23	40	52

From the previous table, it is found that a huge improvement in using BIM in the last 7 years happen. This will reflect the construction industry positively because the improvement in practicing BIM leads to material waste minimization which is considered as the main purpose of this study. The respondents demonstrate that about 53% strongly agree about the government on the right track with BIM while only about 14% strongly agree with this issue in 2016. About 61% of respondents strongly agree that engineers hear more about BIM while it is only about 51% in^32^ strongly agree with this issue. Also about 22% of respondents are strongly agree that the government use BIM in public sector work while only about 7% of respondents strongly agree in 2016. So, it is demonstrated that there is a massive improvement in using BIM in the last 7 years.

### Analysis and discussion of practicing of building information modeling in construction industry

For the first questionnaire, Improvement of practicing Building Information Modeling in Egypt is measured. Some questions of El-Yamany^32^ are studied and analyzed to make a comparison between the usages of BIM from 2016 to 2023. For the second questionnaire, practicing of BIM in construction modeling is measured. Some general questions are asked about the importance of using BIM in construction projects in Egypt. The respondents answers based on scale from 1–5 to clarify their opinions.

Table 3 illustrates the importance and Practicing Index (Pi) of using BIM.

**Table 3:** Importance and Practicing Index (Pi) of using BIM.

Importance of using BIM	Consultant Engineer			Contractor Engineer			Total Respondents		
	Mean	SD	%P_i_	Mean	SD	%P_i_	Mean	SD	%P_i_
Using BIM in design stage	4.39	0.70	88	4.45	0.66	87	4.42	0.67	87
Useful of BIM in residential projects	4.17	0.76	83	4.24	0.74	83	4.20	0.75	83
Useful of BIM in non-residential projects	4.71	0.46	94	4.57	0.55	89	4.64	0.50	92
Useful of BIM in infrastructure projects	4.76	0.45	95	4.71	0.49	91	4.73	0.46	93
Importance of BIM in 3D	4.81	0.39	96	4.84	0.40	97	4.82	0.40	96
Importance of BIM in 4D	4.65	0.50	93	4.63	0.56	93	4.64	0.53	93
Importance of BIM in 5D	4.53	0.61	91	4.47	0.61	89	4.51	0.61	90
Importance of BIM in 6D	3.39	0.87	64	3.32	0.90	56	3.36	0.88	61
Importance of BIM in 7D	3.41	0.88	63	3.01	0.83	51	3.24	0.88	58
Actual practicing of BIM in 3D	4.70	0.48	94	3.58	0.61	72	4.19	0.77	84
Actual practicing of BIM in 4D	4.31	0.63	86	2.84	0.66	43	3.74	0.96	67
Actual practicing of BIM in 5D	3.97	0.73	78	2.72	0.64	40	3.49	0.92	61
BIM improve quantity take-off and estimating	4.49	0.56	90	4.42	0.61	84	4.46	0.58	87
BIM importance in coordination	4.78	0.46	96	4.74	0.44	91	4.76	0.45	94
Engineers hear more about BIM currently	4.68	0.47	93	4.76	0.43	94	4.71	0.45	94
Engineers think BIM is the future of project information	4.75	0.46	95	4.64	0.48	93	4.70	0.47	94
Engineers think the Government is on the right track with BIM	4.27	0.87	85	4.01	0.97	80	4.15	0.92	83
The Government use BIM for public sector work	4.00	0.86	80	4.00	0.97	80	4.00	0.90	58
BIM facilitates modified design or construction methods	4.58	0.53	92	4.57	0.55	91	4.57	0.53	92

From the previous table it is found that about 94% of consultants actually practicing BIM in 3D while about 72% of contractors agree with practicing BIM in 3D. Also it is found that about 86% and 78% of consultants actually practicing BIM in 4D and 5D while only about 43% and 40% of contractors agree with practicing BIM 4D and 5D model respectively. This prove that using BIM in Egypt is up to 5D and consultants use BIM more than contractors. It is also found that about 61% and 58% of total respondents considered that BIM is important in 6D and 7D respectively because it isn’t widely used in Egypt. It is found that 90% of consultant and 84% of contractors considered that BIM improve quantity take-off and estimating. About 96% of consultants and 91% of contractors considered BIM importance in coordination. It is also demonstrated that about 87% of respondents are considered the using BIM in design stage. It is also demonstrated that about 83%, 92% and 93% of respondents considered using BIM in residential, non-residential and infrastructure projects respectively. Also it is found that about 96%, 93% and 91% of consultants agree with the importance of BIM in 3D, 4D and 5D while only about 97%, 93% and 89% of contractors agree with the importance of BIM in 3D, 4D and 5D model respectively. So, the reason for the disagreement between the consultant and the contractor regarding the results is that the contractor does not use BIM and its applications to the same extent that the consultant uses it, and the contractor does not rely on using BIM permanently during implementation on site. It is also found that about 94% of total respondents agreed that Engineers hear more about BIM currently and Engineers think BIM is the future of project information. About 83% of total respondents think the Government is on the right track with BIM and about 58% of total respondents think the government use BIM for public sector work. Finally, about 92% of total respondents agreed that BIM facilitates modified design or construction methods. So, using BIM is very important and should be developed in Egypt to include all project lifecycle and all stakeholders.

## Conclusion

The findings reveal that, based on the first questionnaire, there is a massive improvement in using BIM in the last 7 years in Egypt but using BIM is still limited and not effective in Egypt till now. So it is very necessary for engineers to have knowledge about BIM implementation in order to be qualified to work with BIM. For the second questionnaire, it is found that the number of consultant’s site engineer’s respondents are more than contractors because the usage of BIM is effective in the field of design and consultancy more than using in site. It has been shown that while 72% of contractors agree with using BIM in 3D, 94% of consultants actively do so. Additionally, it was shown that while only roughly 43% and 40% of contractors agreed to the use of the BIM 4D and 5D models, respectively, 86% and 78% of consultants actually use BIM in these two dimensions. This demonstrates that consultants utilize BIM more than contractors do, and that BIM usage in Egypt is on par with 5D. Additionally, it was discovered that due to BIM's limited adoption in Egypt, 61% and 58% of respondents thought that it was crucial in 6D and 7D, respectively. 90% of consultants and 84% of contractors said that BIM improved quantity take-off and estimating, according to the study. Around 96% of 91% of contractors and consultants said BIM was important for coordination. Additionally, it is shown that roughly 87% of respondents thought that BIM is being used throughout the design stage. Furthermore, it is shown that around 83 percent, 92 percent, and 93 percent of respondents thought about adopting BIM in residential, non-residential, and infrastructure projects. Also using BIM is very effective but not used in a wide range. So, it is found that there is a massive improvement in using BIM in the last 7 years. Also, the importance of BIM among consultants is high, and they use it in projects to a greater extent than contractors.

### Recommendations

Monitoring the results of the current study, trying to develop it on a larger scale in construction projects in general, and trying to develop the use of BIM more effectively to reduce the percentage of waste.

## Data Availability

The datasets used and/or analyzed during the current study available from the corresponding author on reasonable request.

## References

[1] Olatunji OJ (2008). Material Wastage Causes: Causes and Their Contributions’ Level.

[2] Garas GL, Anis AR, El Gammal A (2001). Material Waste in the Egyptian Construction Industry.

[3] Shaqour, E. N. & Almashhour, R. T. Causes of building material waste in construction sites in Egypt, Vol. 59, No.1. https://www.researchgate.net/publication/353946793 (2020).

[4] Osama AD, Ahmed AEO, John OE, Bayyati A (2021). Quantifying materials waste in the Egyptian construction industry: A critical analysis of rates and factors. Ain Shams Eng. J..

[5] Reda ELI, El Mohamed G, Hussein AI, Osama AD (2023). Analysis of factors affecting construction and demolition waste safe disposal in Egypt. Alex. Eng. J..

[6] Ahankoob, A., Meysam, S. K., Rostami, R. & Preece, C. BIM perspectives on construction waste reduction. In *Proc. Ann. Conf. of the Management in Construction Research Association* (*MICRA*), *Kuala Lumpur, Nov. 6* (2014).

[7] Eastman C, Teicholz P, Sacks R, Liston K (2008). BIM Handbook: A Guide to Building Information Modeling for Owners, Managers, Designers, Engineers and Contractors.

[8] Eastman, C. M. *et al*. *An Outline of the Building Description System. Research Report No. 50* (Inst. of Physical Planning, Carnegie Mellon Univ, 1974).

[9] Penttila, H. Early architectural design and BIM. In *CAADFUutures ’07* (eds Dong, A. *et al.*) 291–302 (Helsinki University of Technology, 2007).

[10] CRC Construction Innovation (2007). Adopting BIM for Facilities Management. Solutions for Managing the Sydney Opera House.

[11] Swetha SK, Tezeswi TP, Siva MVNK (2020). An assessment of construction waste management in India: A statistical approach. Waste Manag. Res.

[12] Vilventhan A, Ram VG, Sugumaran S (2020). Value stream mapping for identification and assessment of material waste in construction: A case study. Waste Manag. Res.

[13] Wimalasena BADS, Ruwanpura JY, Hettiaratchi JPA (2016). Modeling construction waste generation towards sustainability. ASCE Constr. Res. Congress.

[14] Autodesk. Realizing the Benefits of BIM.4 (2011).

[15] McGraw Hill Construction (2012). The Business Value of BIM in North America.

[16] WRAP UK Construction. BIM (Building Information Modeling) utilization to achieve resources efficiency in construction: Leeds Arena. Basrd on a medium-sized petrol engine-data from Defra/DECC 2011 guidelines to Green House Gas emission factors. https://www.wrap.org.uk/construction (2011).

[17] Hoffman, J. J. Leveraging BIM for steel construction—Current state of integrated processes. In *The Pacific Steel Conference* (*PSSC 2013*), *Singapore, 8–11 October 2013* (2013).

[18] Nawari NO, Chichugova T, Mansoor S, Delfin L (2014). BIM in structural design education. Comput. Civ. Build. Eng. ASCE.

[19] Europe INNOVA. 15 Dec-last update, The STAND-INN HANDBOOK (online) (2008). http://standards.eu-innova.org/Pages/NewsDetail.aspx?id=172 (2011).

[20] Azhar S, Hein M, Sketo B (2011). Building information modeling (BIM): Benefits, risks and challenges. Leadersh. Manag. Eng..

[21] Li, J. *et al.* A project-based quantification of BIM benefits. *Int. J. Adv. Robot. Syst*. 1–13 (2014).

[22] Cheng, J. C. P & Kumar, S. S. A BIM based construction site layout planning framework considering actual travel paths. In *The 31st International Symposium on Automation and Robotics in Construction and Mining (ISARC 2014)* (2014).

[23] Smith, P. Project cost management with 5D BIM. In *29th World Congress International Project Management Association (IPMA) 2015, IPMA WC 2015, 28–30 September–1 October 2015, Westin Playa Bonita, Panama* http://www.sciencedirect.com (2015).

[24] Sheng, X. L., Wei, C. T. & Faris, M. K. 5D building information modelling—a practicability review. In *MATEC Web of Conferences 66*, 00026 (2016), IBCC 2016, 1–7 (2016).

[25] Vitasek, S. & Zak, J. BIM for cost estimation. In *Proceedings of 3rd International Conference on Engineering Science and Technologies. Kosice*. ISBN: 978-80-553-2982-6 (2018).

[26] Vitasek, S. & Zak, J. Cost estimation and building information modeling (BIM) in road construction. In *Proceedings of the Creative Construction Conference (2018), CCC 2018, 30 June–3 July 2018, Ljubljana, Slovenia*. 403–410 (2018).

[27] Hosny S, Nabil Y, Ibrahim A (2022). Causes of reinforced concrete materials waste in construction projects. Egypt. Int. J. Eng. Sci. Technol..

[28] Moradi, S. & Sormunen, P. Integrating lean construction with BIM and sustainability: A comparative study of challenges, enablers, techniques, and benefits. 1471–4175. 10.1108/CI-02-2023-0023. Available on Emerald Insight at: https://www.emerald.com/insight/1471-4175.htm (Construction Innovation Emerald Publishing Limited, 2023).

[29] Almujibah H (2023). Assessment of Building Information Modeling (BIM) as a time and cost-saving construction management tool: Evidence from two-story villas in Jeddah. Sustainability.

[30] Pallant, J. *SPSS Survival Manual: A Step-by-Step Guide to Data Analysis using SPSS for Windows* (2007).

[31] Baxter J, Bartlett PL (2001). Infinite-horizon policy-gradient estimation. J. Artif. Intell. Res..

[32] El-Yamany A (2016). Current practices of building information modelling in Egypt. Int. J. Eng. Manag. Econ..

